# Modeling HIV-1 neuropathogenesis using three-dimensional human brain organoids (hBORGs) with HIV-1 infected microglia

**DOI:** 10.1038/s41598-020-72214-0

**Published:** 2020-09-16

**Authors:** Roberta S. dos Reis, Shilpa Sant, Hannah Keeney, Marc C. E. Wagner, Velpandi Ayyavoo

**Affiliations:** 1 Department of Infectious Diseases and Microbiology, Graduate School of Public Health, University of Pittsburgh, Pittsburgh, PA 15261 USA; 2 Department of Pharmaceutical Sciences, School of Pharmacy, McGowan Institute for Regenerative Medicine, UPMC Hillman Cancer Center, Pittsburgh, PA 15261 USA; 3 Department of Bioengineering, Swanson School of Engineering, University of Pittsburgh, Pittsburgh, PA 15261 USA

**Keywords:** Neurological models, HIV infections, Virus-host interactions, Neurodegeneration

## Abstract

HIV-1 associated neurocognitive disorder (HAND) is characterized by neuroinflammation and glial activation that, together with the release of viral proteins, trigger a pathogenic cascade resulting in synaptodendritic damage and neurodegeneration that lead to cognitive impairment. However, the molecular events underlying HIV neuropathogenesis remain elusive, mainly due to lack of brain-representative experimental systems to study HIV-CNS pathology. To fill this gap, we developed a three-dimensional (3D) human brain organoid (hBORG) model containing major cell types important for HIV-1 neuropathogenesis; neurons and astrocytes along with incorporation of HIV-infected microglia. Both infected and uninfected microglia infiltrated into hBORGs resulting in a triculture system (MG-hBORG) that mirrors the multicellular network observed in HIV-infected human brain. Moreover, the MG-hBORG model supported productive viral infection and exhibited increased inflammatory response by HIV-infected MG-hBORGs, releasing tumor necrosis factor (TNF-α) and interleukin-1 (IL-1β) and thereby mimicking the chronic neuroinflammatory environment observed in HIV-infected individuals. This model offers great promise for basic understanding of how HIV-1 infection alters the CNS compartment and induces pathological changes, paving the way for discovery of biomarkers and new therapeutic targets.

## Introduction

Following systemic infection, human immunodeficiency virus-1 (HIV-1) infiltrates the brain by crossing the blood brain barrier (BBB) through infected monocytes from periphery^1–3^. These infected monocytes differentiate to resident macrophages, release infectious particles to infect and expose permissive bystander resident cells, such as microglia and other glial cells^4,5^. Neurons are not infected^6–8^, yet these cells are most susceptible to dysfunction due to the presence of HIV-1 in the CNS^5,9–11^. It has been proposed that low level of viremia along with inflammatory factors released by infected and/or exposed microglia and macrophages are implicated in selective synaptodendritic damage in neurons in the prefrontal cortex, which slowly evolves to CNS pathology^9–14^. The neuronal dysfunction manifests as impaired cognitive function, a syndrome collectively called HIV-1 associated neurocognitive disorders (HAND), which affects more than 50% of HIV-1 positive individuals regardless of antiretroviral treatment^2,5,15,16^.

Studies to delineate the mechanisms underlying early stages of HIV neuropathogenesis are hampered due to the lack of brain-representative models to study HIV-CNS disease. Although a variety of potential molecular players for HAND have been identified^2,12^, much of our current understanding has been derived from examination of *post-mortem* brain tissue with HIV-1 associated dementia (HAD)^17^. However, the less severe forms of disease, such as asymptomatic neurocognitive impairment (ANI) and mild neurocognitive disorder (MND), currently represent much more common forms of cognitive impairment, and *post-mortem* tissues do not allow the study of early stages of infection and disease progression. *Post-mortem* evaluations have been further supported by observations in the simian immunodeficiency virus (SIV)-infected non-human primates^18,19^ and in vitro experimental studies utilizing two-dimensional (2D) tissue cultures models^20–24^. However, these approaches do not reflect the unique and dynamic features of the human brain physiology and inter-individual differences, notably those including the interaction with a human-specific virus as HIV-1. Therefore, developing an appropriate experimental model with relevant human neuronal cell lineages remains a high priority, since no therapeutic treatments are available to ameliorate the comorbid neurodegenerative disease.

Our strategy to circumvent these limitations is to develop a three-dimensional (3D) human brain organoid (hBORG) model built from human neural progenitor cells (NPCs). Human NPCs have the capacity to differentiate into distinct cell types in the brain and to self-organize to form brain-specific cellular architecture, making them ideal for developing a 3D in vitro brain organoid model and to study neurological diseases^25–27^. However, most of the available protocols for 3D brain organoids require complex and time-consuming protocols^27–31^. Similarly, currently available 3D brain organoids also lack microglia, the essential neuroinflammatory component of the associated pathological events required to accurately model HIV neuropathogenesis^2,4,32^.

Human microglia stem from myeloid precursor cells that originate from the embryonic yolk sac^33,34^. These precursor cells migrate through the bloodstream to infiltrate the developing brain, where they undergo maturation^33,35^. In the healthy adult brain, microglia constitute about 0.5–17% of total cells, depending on the brain region^36^. Any disturbance that affects brain homeostasis such as infection, trauma or altered neuronal activity can elicit rapid and pronounced changes in microglial morphology, gene expression, and functionality that are associated with inflammation^33,34,37^. Microglia and macrophages are the major cell types that are productively infected by HIV-1 in the brain^7,8^. Although little is known about the exact pathological role of microglia in HIV neuropathogenesis, its activation due to HIV-1 infection likely contributes to neurotoxicity observed during HAND^2,12,38^. While infection of astrocytes still remains controversial, astrocytosis is another important event in the HIV-associated CNS pathology^2,39^. Thus, a 3D tri-culture experimental model is critical to investigate the mechanisms of HIV-induced neuropathogenesis; however, such model has not yet been explored for HIV-1 infection to the best of our knowledge.

Here, we have developed a 3D hBORG model using NPCs as precursor cells, which can self-organize and differentiate into major cell types found in the brain, including neurons and astrocytes. We further tested the ability of these hBORGs to support HIV-1 infection as well as to recapitulate the hallmarks of CNS pathology seen in HIV-1 patients by incorporating HIV-infected primary microglia. Our hBORG model displays both neuronal and glial characteristics, where cells self-organize in a complex network. To further model HIV-1 neuropathogenesis, we have successfully engineered a tri-culture system incorporating microglia into hBORGs recapitulating their natural infiltration process^33^. Incorporation of HIV-infected microglia into hBORGs (MG-hBORGs) resulted in inflammatory response and induced damage to neurons and astrocytes, major hallmark features seen in the CNS of HIV-1 infected individuals. Collectively, our results suggest that this novel microglia-incorporated hBORGs (MG-hBORGs) provide a valid brain-representative in vitro model with improved physiological relevance over standard 2D experimental models for investigating the pathogenesis of HIV-1 in the human brain.

## Results

### Optimized mixed culture protocol efficiently drives differentiation of both neurons and astrocytes simultaneously

**Figure 1:**
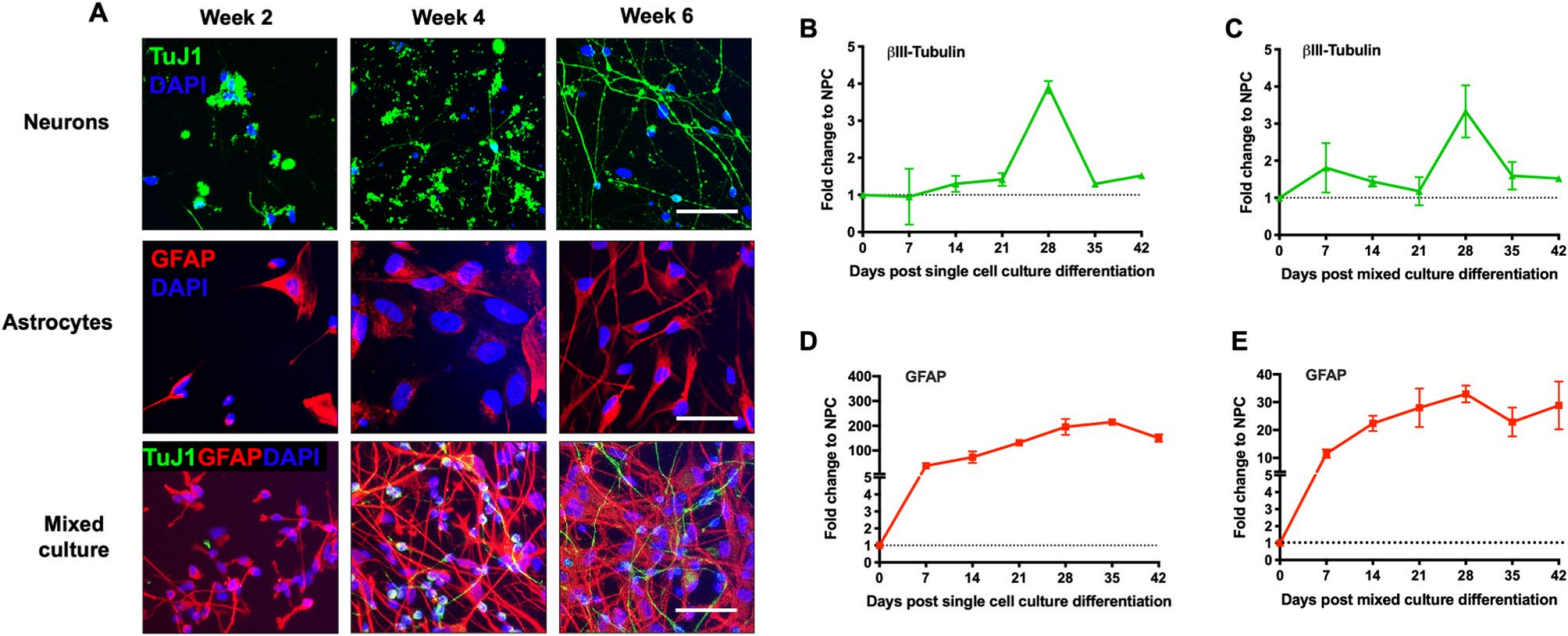
Differentiation and characterization of mixed 2D primary brain cultures. (**A**) NPCs were differentiated into either single culture or mixed cultures according to the protocol described and characterized for expression of cell specific markers by immunofluorescence. Representative images comparing single cell and mixed culture differentiation are depicted. Neurons were stained with βIII Tubulin Tuj1 (green) and astrocytes were stained with GFAP (red) and nucleus with DAPI (blue) at week 2, 4 and 6 post differentiation. Scale bar, 50 μm. **(B**,**C)** Time-course RNA analysis of the neuronal marker βIII Tubulin expression during differentiation in monocultures (**B**) compared to mixed culture (**C**) by qRT-PCR. **(D**,**E)** Time-course analysis of GFAP RNA, astrocyte marker expression during differentiation in monocultures (**D**) compared to mixed culture (**E**) by qRT-PCR. Fold change was calculated by normalizing expression level in undifferentiated NPCs in (N = 3) independent experiments.

NPCs are self-renewing and multipotent cells that can give rise to almost any cell type of developing brain^40^. Here, we tested the generation of 2D mixed brain cultures from NPCs by using the combination of neuronal media and astrocyte differentiation media in comparison with single lineage differentiation media. Immunostaining was performed in mixed brain cultures using specific neuronal (TuJ-1, a βIII-Tubulin epitope) and astrocyte (GFAP) markers along with the single differentiation cultures at week 2, 4 and 6 post differentiation (Fig. 1A). Single neuronal and astrocyte cultures expressed TuJ-1 and GFAP, respectively as early as 2 weeks (Fig. 1A, neurons and astrocytes panels). On the other hand, in mixed cultures, a TuJ-1-positive signal appeared on week 4 and increased by week 6 while a GFAP-positive signal was observed as early as 2 weeks (Fig. 1A, mixed culture panel). Similar results were obtained by assessing mRNA expression of these markers. Comparison of the expression of the neuron-specific cytoskeletal marker βIII-Tubulin between single (Fig. 1B) and mixed culture (Fig. 1C) differentiation protocols indicated that both protocols lead to peak expression of βIII-Tubulin on day 28 in culture (3.9-fold and 3.3-fold change from monoculture and mixed cultures, respectively, compared to NPCs). Expression of the astrocytic marker GFAP increased by 38.8-fold at the initial phase of single culture differentiation (day 7), peaked on day 28 and plateaued afterwards (Fig. 1D). Although less robust, the increase in GFAP expression in mixed culture followed the same trend as single culture (Fig. 1E). Together, our results suggest that the optimized mixed culture protocol is equally efficient as single culture differentiation protocol to drive differentiation of both neurons and astrocytes simultaneously. Moreover, neurons in the mixed culture express markers of mature neurons such as MAP2 (Figure S1A) and markers of synaptic activity including synaptophysin (SYN) and PSD95 (Figure S1B) at week 6, suggesting that our mixed culture differentiation protocol results in mature neurons.

### Human NPCs aggregate to form human brain organoids (hBORGs) upon treatment with mixed differentiation media

**Figure 2:**
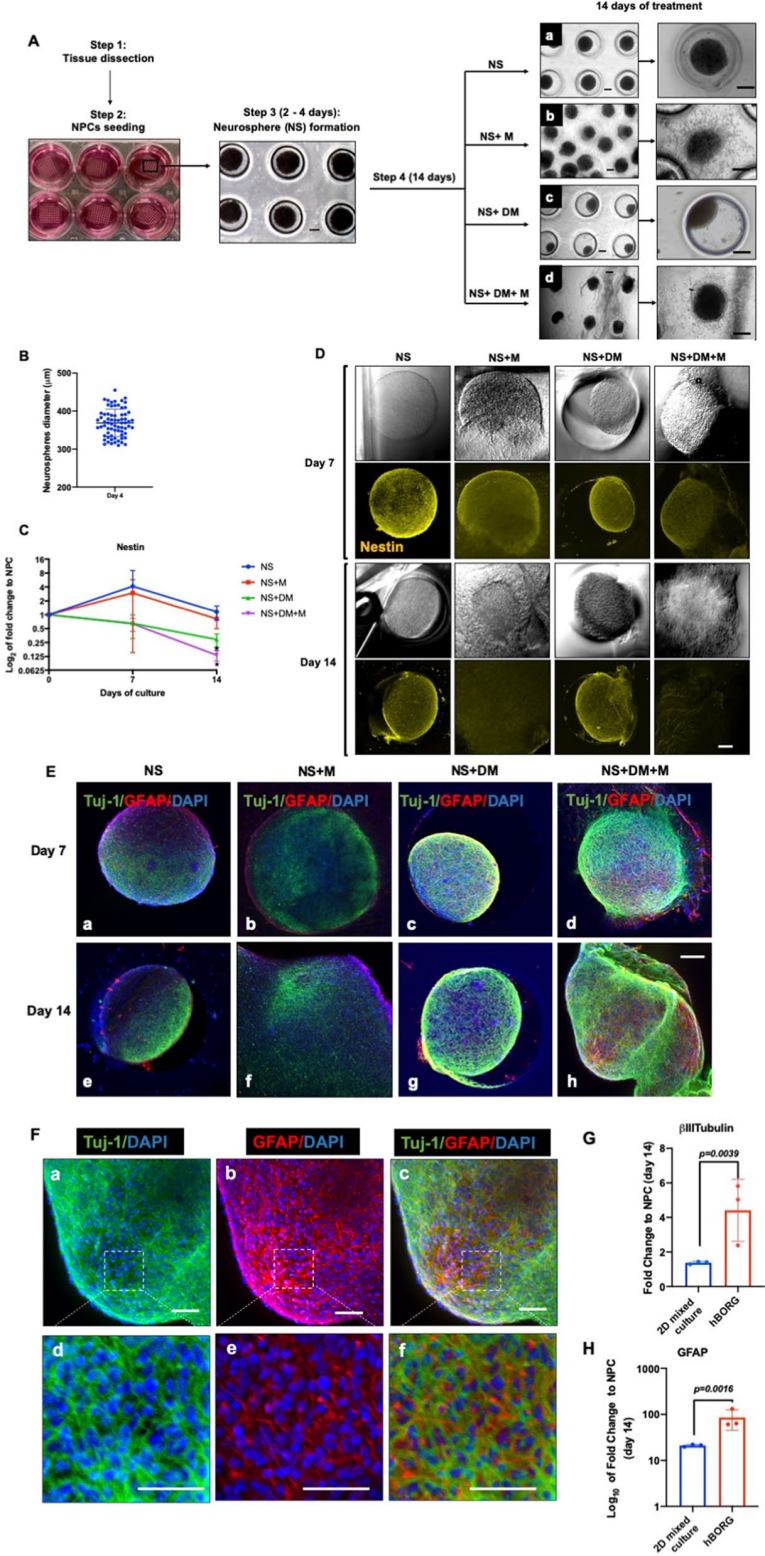
Generation and characterization of size controlled hBORGs from human NPCs. (**A**) Schematics to illustrate the hydrogel devices and experimental design to generate neurospheres from NPCs (step 2). Bright field images show formation of uniform-sized neurospheres in the microwells 2 to 4 days post culture (step 3). Step 4: Phase-contrast images of neurospheres captured 14 days post treatment in different culture conditions: neurospheres in media only (panel **a**), neurospheres in media plus matrigel overlay (panel **b**), neurospheres in differentiation media only (panel **c**), and neurospheres in differentiation media plus matrigel overlay (panel **d**). Scale bar, 100 μm. Higher magnification of resulting organoids are shown at the right panel. Scale bar, 200 μm (**B**) Size distribution of neurospheres on day 4 post seeding, size of multiple neurospheres (N = 70) from 5 different devices were calculated as described in methods and the size distribution is presented. (**C**) Expression of the neuroprogenitor marker, Nestin was measured by qRT-PCR on 14 days post differentiation in different treatments conditions as indicated. NS, neurospheres in culture media; NS + M, neurospheres in culture media and martigel; NS + DM, neurospheres in differentiation media; NS + DM + M, neurospheres in differentiation media with matrigel. Amount of Nestin RNA transcripts in organoids was normalized to undifferentiated NPCs. Statistical significance was determined by unpaired Student’s t-tests, *p < 0.05 of three independent experiments (N = 3). (**D**) Expression of Nestin was assessed by immunofluorescence using anti-Nestin antibody. Representative confocal images validate decreased level of Nestin (yellow). Scale bar, 100 μm. (**E**) Expression of neuronal marker, βIII-Tubulin Tuj-1, (green) and glial marker, GFAP (red) was determined in all four different treatments by immunofluorescence on day 7 and 14 post differentiation. Nucleus was stained with DAPI (blue) Scale bar, 100 μm. (**F**) Expression of neuronal marker, βIII-Tubulin Tuj1 (green) and astrocyte marker, GFAP (red) was measured in neurospheres cultured in differentiation media with matrigel on day 14 post differentiation. Scale bar in panel a, b and c is 50 μm and in panel d, e and f is 100 μm. **(G**,**H)** Expression level of βIII-Tubulin (**G**) and GFAP (**H**) RNA transcripts in hBORGs compared to 2D mixed culture 14 days post differentiation (N = 3). Fold change was calculated by comparing the level of RNA in undifferentiated NPCs. Statistical significance was calculated using unpaired Student’s t-test and the *p* value is indicated in the figure.

Based on the results obtained with our mixed differentiation of brain cells from primary NPCs in 2D monolayers, we next developed a 3D culture platform to generate brain organoids. We adapted a hydrogel microwell platform previously described for generation of uniform size microtumors to generate uniform size brain organoids in a high throughput manner^41–45^. NPCs were seeded onto the hydrogel devices containing multiple 600 μm microwells that enabled formation of aggregates, referred henceforth as neurospheres (NS) within 24–48 h (Fig. 2A). Each 1 × 1 cm^2^ device generated 70–80 NS simultaneously. NS were grown in NPC media until they were compacted (4 days). To determine the size variations of the NS, their diameters were measured 4 days post culture. Overall, the average size of the neurospheres were ~ 369 ± 36 μm (Fig. 2B). Assessing the viability of compact NS indicated that cells were viable at least for 4 weeks (Figure S2). To generate human brain organoids (hBORGs) from these NS, we tested two different protocols for differentiation: culturing NS in mixed differentiation media only (NS + DM, Fig. 2A, panel c) or NS overlaid in matrigel matrix and supplemented with mixed differentiation media (NS + DM + M, Fig. 2A, panel d). To tease out the contribution of the matrigel to cell differentiation, we carried out cultures of NS only as a negative control (Fig. 2A, panel a) and NS overlaid with matrigel supplemented with NPC media (Fig. 2A, panel b). We monitored these four culture conditions for morphological changes for 7–14 days. Results suggest that NS only and NS + DM treatment triggered retraction of NS boundaries (Fig. 2A, panels a and c), whereas, the addition of matrigel with or without differentiation media triggered spontaneous outgrowth of projections from NS resembling neurites within the first 24 h (Fig. 2A, panels b and d, and Figure S3, panels A and B). Continuation of culture for 14 days in differentiation media along with matrigel showed that these neurites appeared to fuse with neurites of adjacent organoids that became tightly interconnected (Fig. 2A, panel d and Figure S3, panels C and D).

To further characterize the differentiation capabilities of the organoids, we first assessed the expression of neuronal progenitor marker, Nestin, both at mRNA and protein levels. Compared to non-differentiation conditions (NS only and NS + M), both differentiation treatments (NS + DM and NS + DM + M) led to a significant decrease (3.4-fold and 7.5-fold, respectively, compared to NPCs) in levels of Nestin mRNA over time (Fig. 2C), suggesting the transition of progenitor cells to differentiated phenotypes. Similarly, a significant reduction of Nestin-positive cells was also observed in NS + M and NS + DM + M at day 7 by Nestin-specific staining, which virtually disappeared at day 14 in NS + DM + M cultures (Fig. 2D) indicating a transition from a proliferating to a post-mitotic state.

Next, to characterize the differentiated cell lineages within the organoids, we stained the organoids from all four groups for specific neuronal (TuJ-1) and astrocyte (GFAP) markers. As expected, qualitative assessment of the staining intensity on day 7 and 14 post treatment exhibited a strong signal for TuJ-1-positive neurons, when NS were cultured with differentiation media (Fig. 2E, panels c, d, g and h). However, distinct neuronal and glial cell populations could be clearly observed only in matrigel embedded organoids treated for 7 days with mixed differentiation media (Fig. 2E, panel d). Additionally, the same treatment (DM + M) led to accumulation of GFAP-positive signal 14 days post differentiation (Fig. 2E, panel h). In contrast, NS cultured in non-differentiation conditions (NS and NS + M) induced less differentiation into TuJ-1-positive neurons by day 7 without further improvement by day 14 (Fig. 2E, panels a, b, e and f). Similarly, lower levels of GFAP+ astrocytes were observed by day 14 in non-differentiation conditions (Fig. 2E, panels a, b, e and f). Immunostaining results also suggested that addition of matrigel may have shifted the ratio of neurons/astrocytes towards neuronal population (Fig. 2E, panel h). Overall, addition of matrigel to the culture containing differentiation media (NS + DM + M) produced the best response in terms of expression of neuronal and astrocytic markers. Higher magnification images of the NS + DM + M-treated cultures revealed an intricate network of neurons and astrocytes (Fig. 2F, a–e) as revealed by zoomed confocal images (Fig. 2F, panel f). To compare the efficacy of simultaneous differentiation of the 2D versus 3D cultures (NS + DM + M treatment), towards neuronal and astrocyte lineages, we quantified βIII-Tubulin and GFAP expression by qRT-PCR at day 14 (Fig. 2G,H). Comparison between 2D mixed cultures and hBORG cultures demonstrated that both neuronal and astrocytic differentiation were promoted significantly (2.9-fold and fourfold, respectively) in 3D cultures, reflecting the ability of 3D microenvironment to promote faster NPCs differentiation in vitro. Taken together, these results suggest that 3D-NPC cultures treated with matrigel and differentiation media displayed decreased NPC stemness, enhanced simultaneous differentiation into both neuronal and astrocytic lineages, and induced spatial organization with intricate cellular networks in each individual organoid while simultaneously generating 70–80 human brain organoids (hBORGs) in one device.

### hBORGs express differentiated and mature cell types and remained viable for at least 25 weeks

**Figure 3:**
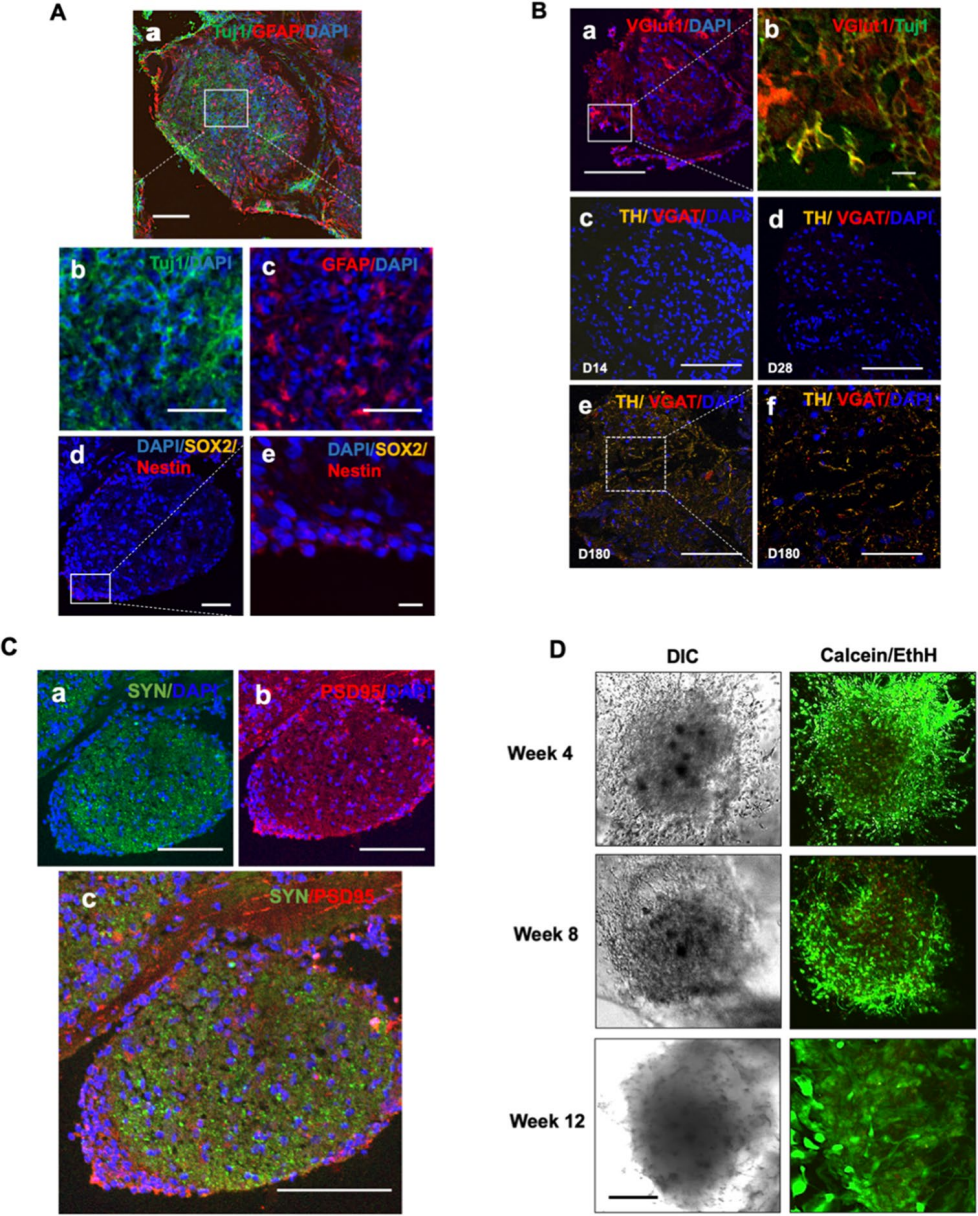
Expression of selected neuronal markers in hBORGs. (**A**) Representative images of comparison of neuronal and glial markers expression by co-immunostaining in hBORGs sections in panel **a**. At day 14 after differentiation, βIII-Tubulin Tuj1 + neurons (panel b) and GFAP + astrocytes (panel c) were identified without a preferential localization. At day 14, few cells were Nestin + /SOX2 + (panel d and e). Scale bar in panel a and d is 100 μm, in panel b and c is 50 μm and in panel e is 10 μm. **(B)** Representative images of neuronal lineage markers. hBORGs sections from day 14 showed that most of differentiated neurons expressed VGlut1 (panel a and b), marker of glutamatergic neuronal lineage. Minimal expression of VGAT, marker of GABAergic neuronal lineage, was observed in hBORGs sections from day 28 and was maintained up to day 180 (panels c, d and e), whereas the expression of tyrosine hydroxylase (TH), marker of dopaminergic neuronal lineage, is evident only at day 180 (panels e and f). Scale bar in panels a, c, d and e is 100 μm and in panels b is 10 μm and f is 50 μm. (C) Expression of synaptic markers synaptophysin (SYN), PSD95 were observed as early as day 14 of differentiation (panel a-c) (**D**) Cell viability in hBORGs was assessed by live/dead assay followed by confocal microscopy. Images represent live cells (green) and dead cells (red) from one of the representative experiments (N = 3). Scale bar is 100 μm.

To characterize differentiation and maturation process of cells in the organoids, hBORGs were harvested on 14, 28 and 180 days, sectioned and sections were stained for specific neuronal and glial markers. Neurons were positive for the neuron-specific cytoskeletal marker β-III-tubulin (Tuj1) (Fig. 3A, panels a and b). Further confocal microscopy analysis of sections revealed that hBORGs protocol resulted in the generation of dense neural networks (Fig. 3A, panel b, and Figure S4A). GFAP + cells constituted 39% of all DAPI+ nuclei (Fig. 3A, panel a and c, and Figure S4A) and can be identified among neurons in direct proximity of neuronal bodies. Additionally, hBORGs harvested on day 28 (Figure S4B) and day 180 (Figure S4C) stained for Tuj1 and GFAP also demonstrated consistent cytoarchitecture over time. Although no signal for the neuroprogenitor marker, SOX2 (Sex-determining region Y-box 2) was observed at day 14, few Nestin positive cells were observed in the most superficial layer of hBORGs (Fig. 3A, panels d and e) suggesting loss of stem cell identity and enhanced differentiation towards mature brain organoids.

Since HIV neuropathogenesis is generally known to affect glutamatergic circuits^46–48^, we sought to evaluate the presence of glutamatergic neurons in the hBORGs by assessing the expression of vesicular glutamate transporter 1 (VGLUT1) (Fig. 3B, panel a) on day 14. Majority of differentiated neurons (Tuj1+) are VGLUT1 positive (Fig. 3B, panel b), consistent with a glutamatergic lineage identity. Furthermore, tyrosine hydroxylase (TH), that is exclusively expressed in dopaminergic neurons, was observed on day 180, suggesting that this neuronal subtype is spontaneously generated in longer term culture (Fig. 3B, panels c–f). In contrast, minimal expression of vesicular GABA transporter (VGAT) over time confirmed that a small population of differentiated neurons in hBORGs were GABAergic (Fig. 3B, panels c, d and e).

Next, to test the majority of hBORG- glutamatergic neurons, we evaluated the expression of pre-synaptic synaptophysin (SYN) and post-synaptic PSD95 markers (Fig. 3C, panels a and b). Indeed, colocalization of SYN and PSD puncta is suggestive of synaptic network connectivity (Fig. 3C, panel c). Finally, we assessed the viability of hBORGs, which is important to validate the applicability of this model for further experiments. hBORGs were stained with Calcein AM to detect live cells and Ethidium homodimer to detect dead cells at different time points (Fig. 3D). With appropriate maintenance, we observed that hBORGs are viable for more than 12 weeks with minimal cell death as indicated by live/dead staining.

### HMC3 microglia can be incorporated into hBORGs to mimic multicellular crosstalk observed in HIV-1 neuropathology

**Figure 4:**
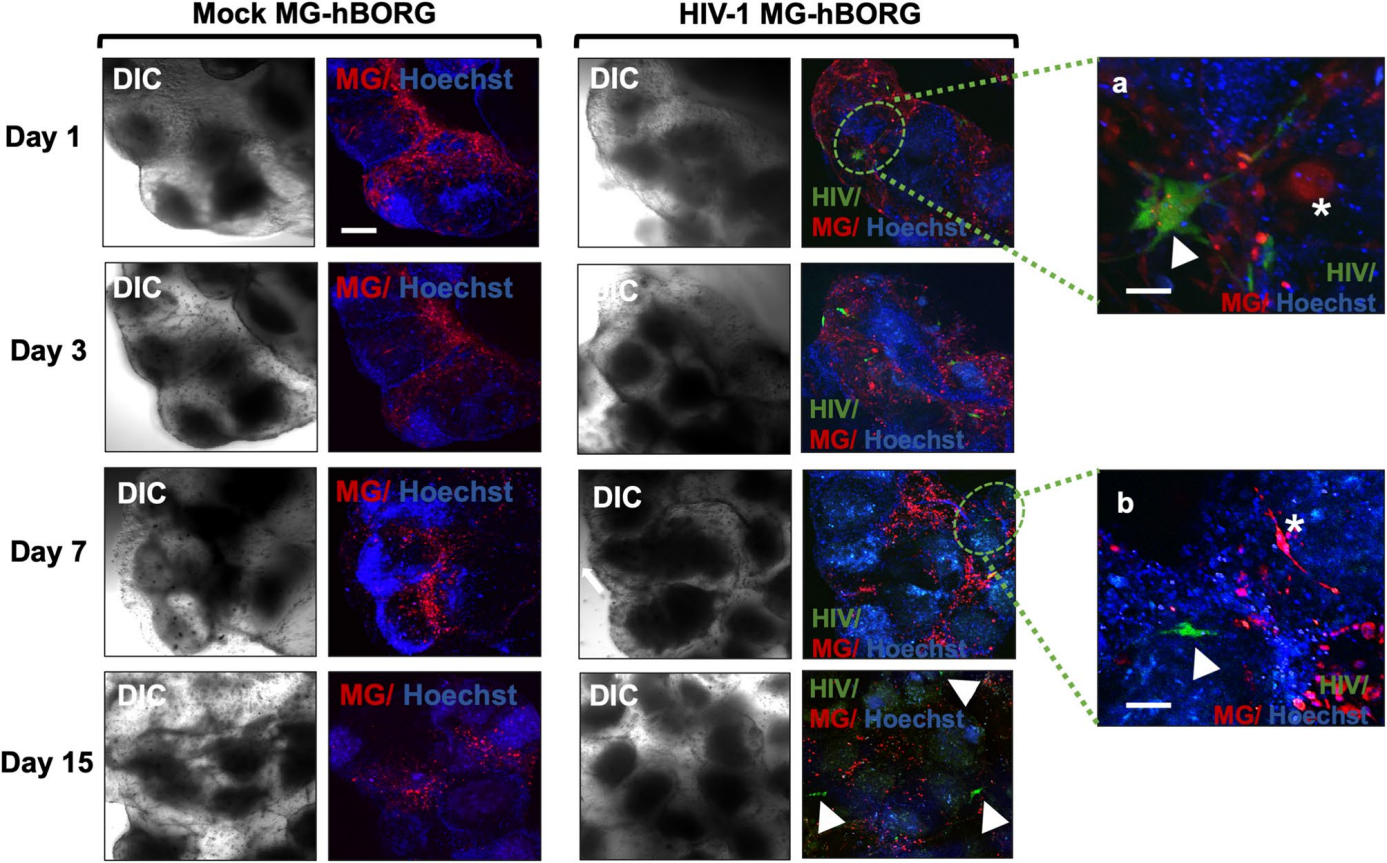
Incorporation of HMC3 human microglia into hBORGs. HMC3 microglia (1 × 10^6^ cells) were infected with HIV-1 (green) or mock-infected, membrane-labeled (red) and were added to hBORGs labeled with Hoechst-stained (blue). After 24 h, microglia incorporated-hBORGs were transferred to a new plate and maintained for an additional 15 days for further analyses. White arrowheads point to HIV-infected microglia (green) in insert a and b, and White asterisks in insert a and b point to uninfected microglia (red). Scale bar is 200 μm and 50 μm in inserts.

The primary focus of developing the hBORG model is to study HIV-1 neuropathogenesis in a human representative system. An important component of HIV-1 neuropathology is the presence of virus-infected human macrophages and microglia, as observed in *post-mortem* brain tissues^49–51^. Thus, we next incorporated microglia into the hBORGs as shown in Fig. 4. Immortalized human microglial cells (HMC3) were infected with neurotropic HIV-1 NL(YU2-Env)-EGFP reporter virus in 2D cultures. Three days post infection, ~ 30% of HMC3 cells were infected (EGFP+) as detected by microscopy. To mimic the in vivo conditions, we incorporated mock- or HIV-infected HMC3 cells by placing them on top of the hBORGs cultures on day 15 post differentiation. Attachment of microglia to hBORGs and migration were monitored for another 15 days using confocal microscopy. Results indicate that by 24 h post incorporation, more than 50% of microglia attached to the hBORGs (Fig. 4) and both infected (green, arrowhead) and uninfected (red, asterisk) microglia continued to infiltrate into the hBORGs from day 1 (Fig. 4, insert a) to day 7 (Fig. 4, insert b).

Although microglia infiltration appeared most abundant surrounding the hBORGs (Fig. 4) by day 3, many cells migrated into the hBORGs layers and were completely embedded into it, as observed by z-stack reconstruction (Supplementary movie 1). These results suggest that both infected and uninfected microglia rapidly respond by migrating into hBORGs similar to the migration of the immune cells from periphery into the CNS.

### Human primary microglia incorporated into hBORGs support HIV-1 replication and alter the cytokine expression levels

**Figure 5:**
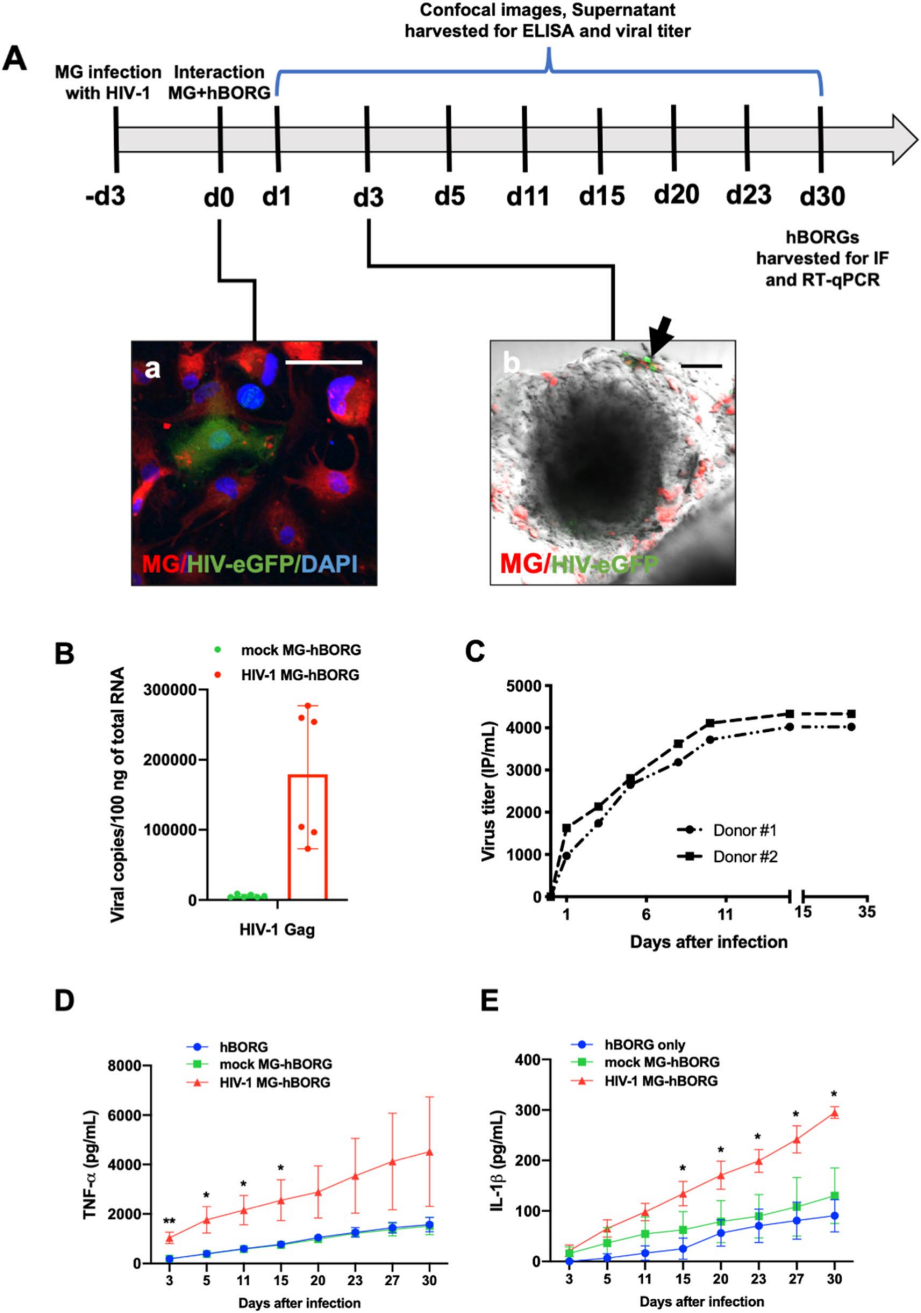
Human adult primary microglia recapitulate engagement with hBORGs, supports HIV-1 infection and produces inflammatory cytokines. (**A**) Schematic diagram of the experimental design is depicted. Primary adult brain microglia (0.5 × 10^6^ cells) were infected with HIV-1 (panel a, green) or mock-infected, membrane-labeled (red) and were added to hBORGs for overnight. Scale is 100 μm. After 24 h, microglia incorporated-hBORGs were transferred to a new plate and maintained for an additional 30 days for further analyses. Image (panel b) depicts the infiltration of primary adult brain microglia into hBORGs. Infected and uninfected microglia were membrane-labeled (red), incorporated into hBORGs and imaged by confocal microscopy. HIV-infected microglia (green, indicated with black arrow) and uninfected microglia (red) was incorporated in hBORGs on day 1 post coculture (panel **b**). Scale bar is 100 μm. (**B**) RT-qPCR assessment of HIV-1 gag mRNA copies in primary MG-hBORGs at day 30 post infection (N = 3). (**C**) Representative cumulative HIV-1 virus titer in supernatants from HIV-infected MG-hBORGs where MG-hBORGs were developed using NPCs from two different donors. Kinetics of cumulative levels of (**D**) TNF-α and (**E**) IL-1β released from HIV-1 infected, mock-infected MG-hBORGs and BORGs without microglia during the course of infection measured by ELISA (N = 4). **p < 0.01, * *p* < 0.05.

We next investigated whether HIV-1 infected microglia incorporated hBORGs (MG-hBORGs) model can recapitulate some of the hallmarks of the HIV-1 CNS pathology in humans. Although the immortalized microglia cell line used in our studies demonstrated the physiological characteristics typical of human microglia such as migration into the hBORGs (Fig. 4), viral replication, and secretion of TNF-α and IL-1β (Figure S5), these cells limited our studies to up to 15 days due to their rapid proliferation. To circumvent this limitation and to establish clinical relevance to our model, we incorporated primary human microglia from post-mortem adult human brain (infected or mock) into two independent sets of hBORGs following the protocol summarized in Fig. 5A. First, we incorporated primary microglia into hBORGs and imaged the infected (green) and uninfected (red) microglia (Fig. 5A, panel a). The primary microglia infiltrated into hBORGs as early as 3 days (Fig. 5A, panel b) similar to the activated phenotype observed for HMC3 cells (Fig. 4). HIV-1 transcriptional activity was investigated by quantification of HIV-1 *gag* mRNA copy number in organoids through qRT-PCR (Fig. 5B). Viral RNA transcripts were detectable in the two MG-hBORGs samples at day 15 post-infection (median 179,000 copies per 100 ng of RNA; range 73,000–277,000 copies per 100 ng of total RNA) suggesting high viral replication in organoids containing microglia. These results were further supported by the assessment of the viral titer in the conditioned media by measuring the infectious particles released into the supernatant. Infectious virions were readily detected in the supernatants of HIV-1 infected MG-hBORGs as early as 1 day post incorporation and increased progressively throughout 30 days culture (Fig. 5C) suggesting that MG-hBORGs supported active viral replication with potential to spread the infection to other target cells.

Activated microglia and macrophages associated with HIV-1 neuropathogenesis are known to release various proinflammatory molecules. Among them, tumor necrosis factor (TNF-α) and interleukin-1 (IL-1β) play a central role in neuroinflammation^52–54^. Hence, we measured cumulative levels of TNF-α and IL-1β in supernatants from MG-hBORGs by ELISA. Very low or below detectable levels of TNF-α and IL-1β were observed in hBORGs only and mock-infected MG-hBORG groups, as observed with HMC3 (Figure S5), whereas HIV-1 infected MG-hBORGs showed significantly higher levels of these two cytokines (Fig. 5D,E). TNF-α levels were significantly elevated by 5.7-fold (1,040 ± 227.4 pg/mL) in HIV infected MG-hBORGs compared to mock-infected (196.8 ± 48.7 pg/mL) at early infection (day 3). TNF-α levels continued to increase up to 15 days after incorporation of infected MGs (Fig. 5D), which directly correlated with the viral replication (Fig. 5C). In contrast, increase in the IL-1β secretion occurred at later time points (after day 15 post MG incorporation) as reported by us in monocyte-derived macrophages earlier^55^. IL-1β secretion was enhanced by 2.2-fold (134.5 ± 24.3 pg/mL) in HIV-1 infected MG-hBORGs compared to mock MG-hBORGs (62.4 ± 36.2 pg/mL) on day 15 (Fig. 5E) and remained elevated up to 30 days of co-culture. The same trend was observed in the HMC3 incorporated organoids, that is, increased release of TNF-α and IL-1β (Figure S5). Together, these results confirm that our hBORGs are amenable for integration with primary adult human microglia, support chronic HIV replication and recapitulate the neuroinflammatory milieu that is observed in HIV-1 infected brain.

### MG-hBORG model mimics HIV-1 CNS pathology signatures reported in *post-mortem* brain tissue of HIV-infected individuals

**Figure 6:**
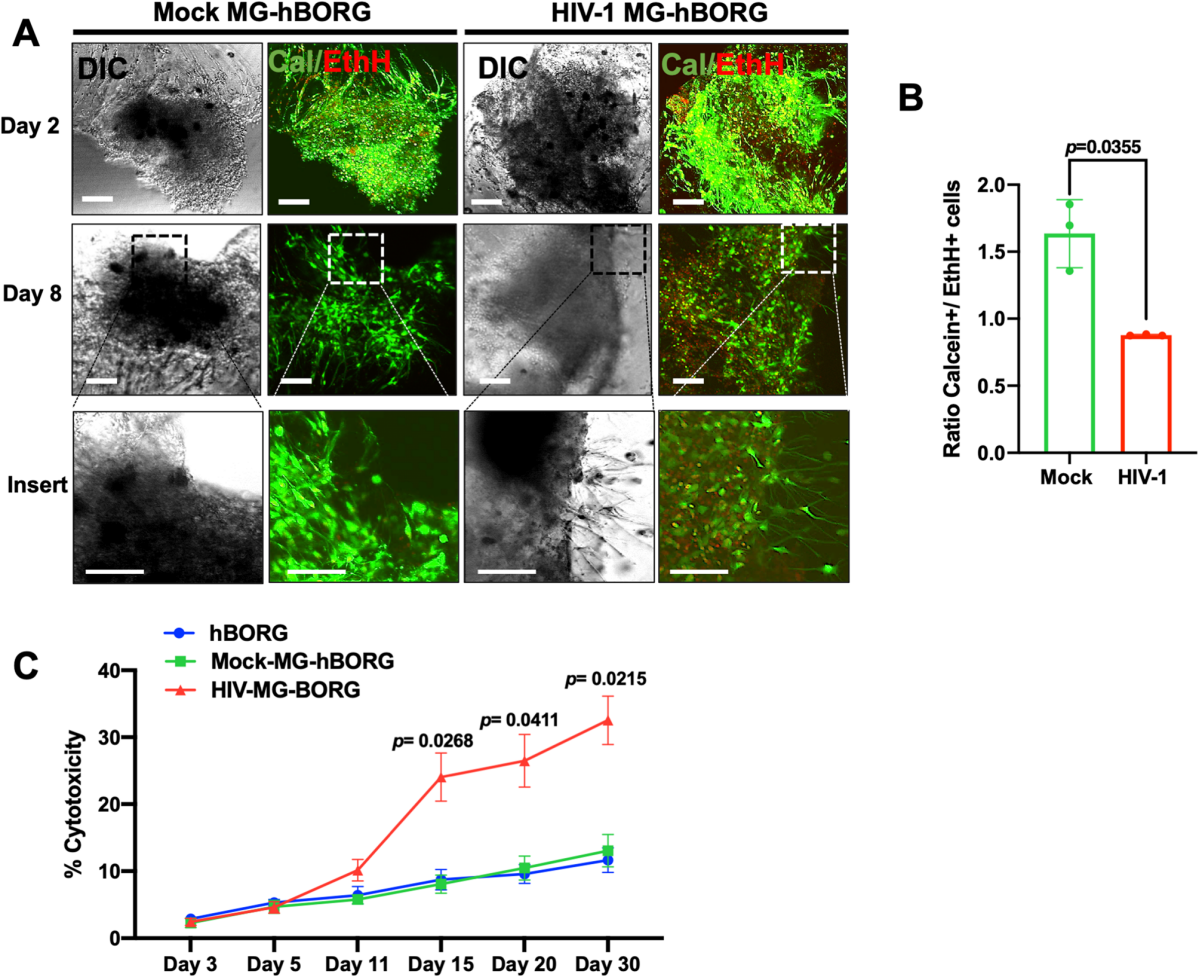
Characterization of viability and cytotoxicity induced by HIV-1 in primary MG-hBORG model. (**A**) Mock-infected and HIV-infected MG-hBORGs were stained with LIVE/DEAD Cell imaging kit to determine the live and dead cells on days 3 and 8 post microglia incorporation. Calcein stains live cells (green) and ethidium homodimer stains nuclei of dead cells (red). Scale bars is 100 μm. **(B)** Calcein and ethidium homodimer positive cells were counted on day 8 and the corresponding ratio of live (calcein +) and dead (EthH +) cells were calculated. (**C**) Cytotoxicity induced by HIV infection was quantified in the supernatant of the HIV-infected, mock-infected MG-hBORGs and hBORGs by measuring LDH activity and % toxicity was determined (N = 3).

We first sought to evaluate whether virus replication and chronic inflammatory condition in infected primary MG-hBORGs would have any effect on the viability of cells in hBORGs. To accomplish this goal, we assessed cell death in MG-hBORGs by staining with Calcein AM (live, green) and Ethidium homodimer (EthH, dead, red) (Fig. 6A). In mock-infected MG-hBORGs, minimal level of cell death was detected; whereas, HIV-1 infection gradually decreased the proportion of Calcein+ cells to the EthH+ cells in infected MG-hBORGs (Fig. 6B). The results of viability from image analyses were further supported by measuring lactate dehydrogenase (LDH) released in the conditioned media from hBORGs-only cultures in comparison to both mock and infected groups as an indicator of cell injury (Fig. 6C). Interestingly, incorporation of mock-infected microglia did not alter the basal level of cytotoxicity in hBORGs cultures. Notably, incorporation of HIV-1 infected primary microglia into hBORGs exhibited a fivefold increase in cytotoxicity as early as day 11 of co-culture compared to mock-infected hBORGs (Fig. 6C). Cytotoxicity peaked at day 15 of co-culture, exhibiting a sixfold increase correlated with viral expansion (Fig. 5C) and suggesting potential loss of either neurons and/or astrocytes in HIV-1 infected MG-hBORGs.

**Figure 7:**
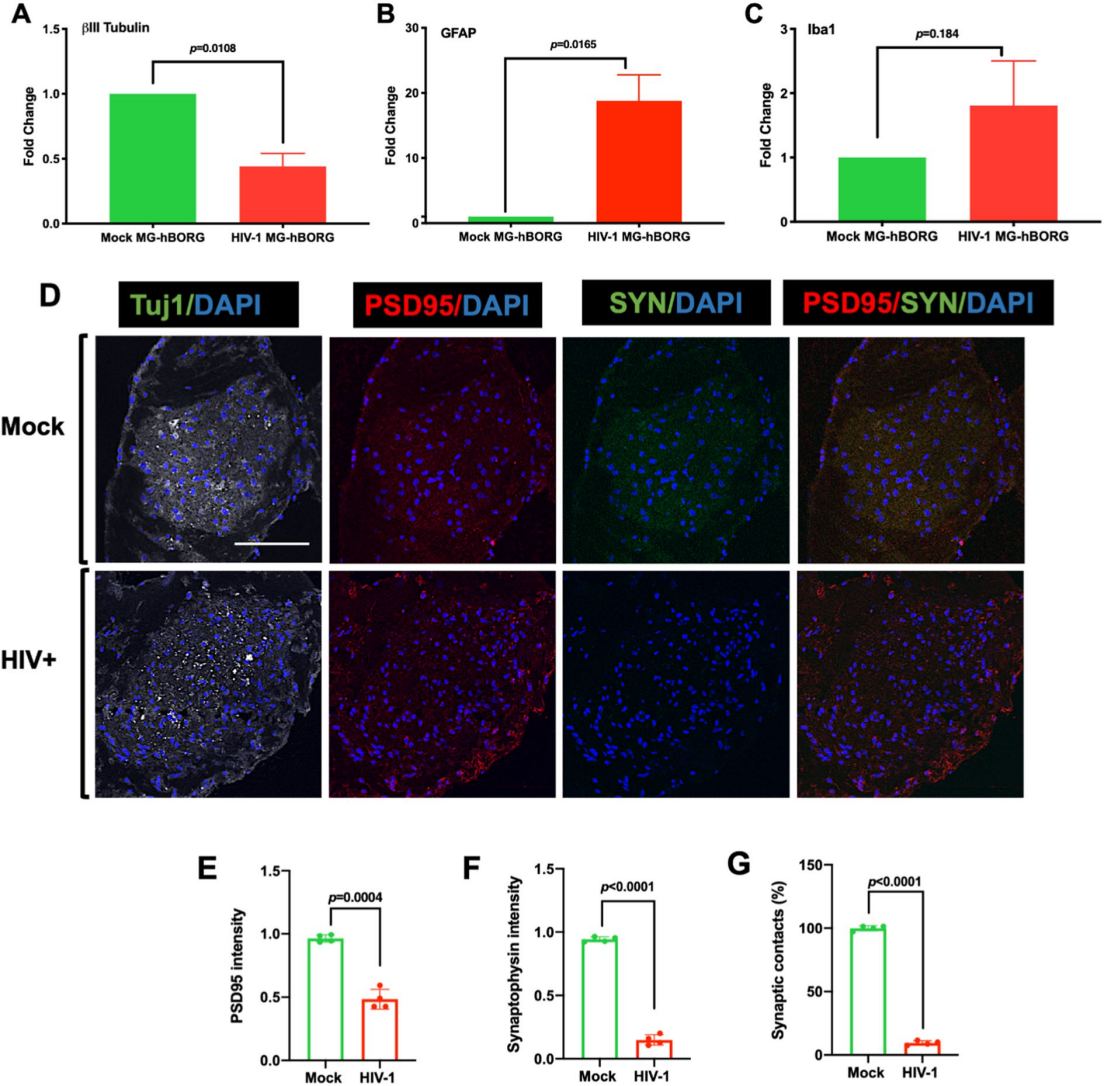
HIV-1 infection of MG-hBORGs causes reactive astrocytosis, decreased synaptic density, and neurodegeneration. Expression levels of βIII-Tubulin (**A**), GFAP (**B**) and Iba1 (**C**) in HIV-infected and mock-infected MG-BORGs were assessed on day 15. Fold change in HIV-infected MG-hBORGs was calculated using mock-infected MG-hBORGs as 1 (N = 3). (**D**) Immunostaining of sections of MG-hBORGs for Tuj1(gray), PSD-95 (red) and SYN (green) were used to stain synapses (PSD95/SYN merged) in viable neurons in mock and HIV-infected organoids. Mean intensities PSD-95 (**E**) and SYN (**F**) were normalized to Tuj1 (maximum intensity). (**G**) The percentage of synaptic contacts (PSD-95/SYN co-localization intensity) in HIV-infected MG-hBORGs was calculated using the intensity of synaptic contacts in mock-infected MG-hBORGs as 100% (N = 4). Statistical significance was calculated using unpaired Student’s *t* test.

Since neurons are the most susceptible cells to damage due to HIV-1^10,11^, we tested whether the expression of neuronal marker βIII-Tubulin is altered in infected MG-hBORGs. Indeed, we observed 2.3-fold decrease in the βIII-Tubulin mRNA by qRT-PCR (Fig. 7A), indicating neuronal loss in infected MG-hBORGs compared to mock-infected MG-hBORGs tested by day 15 post microglia incorporation. In contrast to the neuronal marker expression, the astrocyte marker (GFAP expression) is significantly increased by 18-fold in infected MG-hBORGs compared to mock-infected MG-hBORGs (Fig. 7B) suggesting presence of astrocytosis. However, the expression of the microglial marker, (Iba1) was not significantly affected in HIV-infected microglia containing hBORGs (Fig. 7C). Taken together, these results confirm that HIV-1 infection enhances loss of neurons and astrocytosis in hBORGs. Indeed, reactive morphology of astrocytes together with neurodegeneration are hallmarks of HIV-associated neuropathology in patients with severe HAND^56^. To assess the synaptic damage due to HIV-infection in viable neurons, we further examined the intensity of staining of PSD-95 and Synaptophysin (SYN) immunolabeled puncta (Fig. 7D) on day 30 of MG-hBORGs culture and plotted the mean intensity of each puncta as a fraction of Tuj-1 maximum intensity (Fig. 7E,F). We observed a significant decline of 1.9-fold and 6.7-fold in PSD95+ and SYN+ areas, respectively, in the HIV-MG-hBORGs compared to mock-MG-hBORGs. The higher reduction in the pre-synaptic marker (SYN) may indicate that the pre-synaptic terminals are more susceptible to and hence, are preferentially compromised in HIV-1 infection. To consolidate this finding, we performed image analysis of synaptic contacts between PSD95+ and SYN+ neurons by assessing the mean intensity of the co-localized PSD95/SYN area in HIV-1 infected MG-hBORGs in comparison with the mock-infected MG-hBORGs (Fig. 7G). Indeed, we observed a significant 10.6-fold decrease in the mean co-localization intensity of PSD95/SYN stained puncta in HIV-MG-hBORGs than mock-MG-hBORGs, further suggesting loss of synaptic integrity. Altogether, our data demonstrate the physiological relevance of the MG-hBORG system to study HIV-1-neuropathogenesis in vitro, and future applicability of this model to greatly improve our knowledge of mechanisms underlying initiation and progression of HIV-1-neuropathogenesis.

## Discussion

To study HIV-1 neuropathogenesis, we developed a 3D in vitro brain organoid model derived from human neuroprogenitor cells that recapitulates the neurodegenerative microenvironment of human CNS pathology. We developed an efficient 3D culture system using a hydrogel microwell platform, which enables high throughput production of size-controlled neurospheres that can be differentiated into neurons and astrocytes to form multiple brain organoids simultaneously. We further incorporated HIV-1 infected microglia and demonstrated the utility of our system for modeling the major hallmarks of HIV-1 neuropathology.

Studying neurodegenerative diseases in vitro is challenging due to the complex nature of CNS biology involving multiple differentiated cell lineages^25^. Attempts have been made to generate brain organoids using human induced pluripotent stem cell-derived neural stem cells (hiPSCs- derived NSCs) presenting remarkable progress in this emerging field^28,57–61^. Despite the fact that these systems require expensive techniques with complex protocols that are time-consuming, hiPSCs can be easily acquired, and cultured in vitro to study human diseases. On the other hand, genomic instability and variability in neural differentiation capacity have been reported and may compromise the functionality of iPSC-derived systems^62,63^. Recently, other protocols have been established using human neuroprogenitor cells (NPCs) such as NTera-2 and ReN cells^64,65^. NPCs are immortalized cell lines that have been modified to expand indefinitely and may differ from those found in the in vivo setting. Primary NPCs isolated from embryonic human brain tissue present a limited life span in culture. Studies may be hampered by limited tissue acquisition, and donor variability may pose a drawback. Additionally, fetal brain-derived primary NPCs should be collected within 18–20 weeks of gestation, since extended gestation period (within 18–20 weeks) may result in altered phenotypes and differentiation. Nevertheless, these cells have traditionally been used in 2D culture system, demonstrating efficient differentiation into functional neurons or glial cells and capturing the true heterogenicity akin to in vivo models^20,66^. Hence, we chose fetal brain derived primary NPCs to develop hBORG model in this study.

We first employed a methodology to promote 2D mixed brain differentiation using embryonic NPCs by combining elements of already existing protocols to minimize preparation time and complexity^20,66,67^. Next, we generated uniform size 3D neurospheres to be differentiated into neurons and astrocytes by employing the same customized mixed differentiation media. It has been shown that NPCs respond to the stiffness or resistance of the substrate in which they are embedded, directly affecting their differentiation to neural cells^68^. In our system, the mechanical support was mimicked by the addition of matrigel that greatly improved our 3D system and hBORGs expressed neuronal and astrocytic markers in half of the time compared to the 2D mixed culture system.

Many of the available protocols are entirely focused on neurons and astrocytes and lack microglia or another inflammatory component^26,28,31^. In contrast, we incorporated microglia into mature hBORGs mimicking invasion of microglia into the CNS during neurodevelopment^33^. First, we incorporated the immortalized microglial cell line HMC3 in our model to standardize our system. To mimic the adult HIV patient’s brain, we then tested the incorporation of human adult microglia isolated and cultured from *post-mortem* brain into hBORGs. Abud et al.^57^, Muffat et al.^69^ and Abreu et al.^70^ recently reported incorporation of immortalized or iPSC-induced microglia as inflammatory source in their models. However, none of these studies are reported in the context of HIV-infection, and primary human brain microglia were not included in these studies. Our study, on the other hand, is the first to report incorporation of HIV-infected primary human microglia into human brain organoids to more accurately recreate the human physiological microenvironment. Indeed, our results show that HIV-infected microglia can be incorporated into hBORGs that further support viral replication.

Another common and important feature associated with HIV neuropathology is the release of inflammatory mediators^4,32,39^. Microglia have been implicated as major producers of TNF-α in neurodegenerative diseases and CNS injuries^71,72^. Not surprisingly, HIV infection rapidly induced TNF-α release in our MG-hBORG system, which was directly correlated with the extent of virus replication. This finding is consistent with the early findings conducted in *post-mortem* tissue from HIV-infected individuals correlating both, viral replication and TNF-α transcription^73^. Similarly, IL-1β is also elevated in the CNS during HIV-1 infection^52^. In our study, we observed similar IL-1β release from HIV-1-infected MG-hBORG over the course of the culture consistent with previous work of our group showing variation of IL-1β expression and release by monocyte-derived macrophages throughout the course of HIV-1 infection^55^. Of particular importance was the observation that IL-1β concentration increases with the viral production, suggesting that viral replication directly correlates with the release of this pro-inflammatory cytokine. Consistent with this assumption, Mamik and colleagues have shown that exposure to recombinant Vpr protein induced transcription and release of IL-1β in human microglia in a dose-dependent manner^74^.

It is possible to speculate that other pro-inflammatory cytokines released by infected cells can provoke an additive effect and exacerbate the inflammatory responses of the entire system regardless the level of infection. It is reported that astrocytes are infected by HIV-1 in vivo likely through cell-to-cell contact^75^. However, we found no evidence of astrocyte infection during the period of study in our system based on image analysis of the entire organoid. Although astrocytes are unlikely to be major contributors of IL-1β within the brain, it is important to highlight that the contribution of astrocyte activation/infection to IL-1β peak release remains to be clarified^52^. These cells outnumber microglia in the brain by tenfold and have been shown to respond to LPS-activated microglia increasing TNF-α and IL-1β expression and release leading to a neurotoxic function^76^. Nevertheless, ability of our MG-hBORG model to detect changes in TNF-α and IL-1β release in the conditioned media provides a significant advantage of our tri-culture system over most of the 2D cultures or current brain organoids devoid of microglia and prompts further investigation on the inflammatory response to HIV-1 infection in brain.

Neurotoxic soluble factors released by activated/infected microglia have been shown to contribute to neurodegeneration through different pathways^77–80^. In our model, we observed decreased neuronal viability upon HIV-1 infection, which is in agreement with previous observations in HIV-infected *post-mortem* brain^4,5,17^. Although we have not determined the mechanisms involved, our ability to generate an organoid model that recapitulates HIV-1 neuropathology certainly enables the extended studies of the pathogenic cascade that culminate in neuronal damage.

Although it was not in the scope of this study to investigate a more extensive secretory profile, full examination of the conditioned media would broaden our knowledge in HIV-induced neuroinflammation. In addition, modulation of microglial responses is a potential therapeutic approach for treatment of HIV-1 neuropathology and other neurodegenerative diseases^81^. Thus, future studies with infected MG-hBORG may give insights into the time course of microglial activation and polarization as well as unravel new molecular players that could be potentially targeted for therapies. Although not specifically studied in this report, the HIV-infected MG-hBORG model provides a physiologically relevant human-specific experimental system to further study the dynamics of viral latency and persistence in the absence or presence of antiretrovirals. It remains to be investigated if the cell damage observed in our study can be attenuated through suppression of viral replication. The effects of the combined antiretroviral therapy (cART) correlate with a decrease in the prevalence of HIV-associated dementia^82^; however, high persistent rates of mild to moderate neurocognitive impairment is observed in individuals under cART regimen^5^. Detailed mechanistic studies on synaptodendritic damage by using MG-hBORG system in the presence of ART drugs deserves further investigation and may provide important insights.

Overall, hBORGs provide an alternative and physiologically relevant experimental model for investigating host-viral interactions and to assess molecular mechanisms underlying the progression of neuropathogenesis and the development of HAND. To the best of our knowledge, this is the first study to model HIV-1 neuropathology using hBORGs along with HIV-infected primary microglia. Our tri-culture model addresses key pathological features that are associated with neuroinflammation by HIV-1, defined by the presence of activated microglia, reactive astrocytes and release of pro-inflammatory cytokines. The proposed model has great potential to serve as a human representative 3D model to boost our current knowledge about the molecular dynamics of HIV neuropathogenesis and its progression.

## Methods

### Isolation, culture of NPCs and differentiation of neurons and astrocytes

Primary human NPC cultures were adapted from the method developed by Hammond et al.^20^ and Lu et al.^66^. Human fetal cortical tissue (gestational age of 18–20 weeks) provided by our collaborator, Dr. Moses Bility (University of Pittsburgh), were obtained from medically or elective indicated termination of pregnancy through Magee-Women’s Hospital of UPMC via the University of Pittsburgh, Health Sciences Tissue Bank. Written informed consent of the maternal donors was obtained in all cases, under IRB of the University of Pittsburgh guidelines and federal/state regulations. The use of human fetal cells to build human brain organoids was reviewed and approved by human subjects institutional review board (IRB) of the University of Pittsburgh, in accordance of regulations of Declaration of Helsinki. Briefly, fetal cortical tissues were mechanically dissociated by pipetting. The tissue homogenate was passed through a 40 μm strainer to isolate single neuroprogenitor cells (NPCs). Cells were counted and plated on Poly-D-Lysine/Laminin-coated plates and kept in defined 2D differentiation media: Knockout DMEM/F12 (Invitrogen) supplemented with B27 plus (50× , Invitrogen) in serum-free media for neuronal differentiation or 1% FBS for astrocyte differentiation. For differentiation of mixed brain culture, we combined both B27 plus and 1% FBS in the basal media for culturing. Six weeks post culturing on tissue culture plates, more than 90% of the cells were differentiated and exhibited mature neuronal and/or astrocytic differentiation.

### Fabrication of hydrogel microwell arrays

Non-adhesive hydrogel microwell arrays containing 70–80 microwells per 1 × 1 cm^2^ were microfabricated using polyethylene glycol dimethacrylate (PEGDMA, 1000 Da, Sigma) and polydimethyl siloxane (PDMS) molds as described previously^41–45^. Each microwell was 600 µm in diameter and 600 µm in depth. First, PDMS molds were fabricated as described below. A prepolymer silicone elastomer base solution and curing agent were combined in a ratio of 10:1 (Sylgard 184; Dow Corning Corporation). After removal of bubbles by degassing, the mixture was poured onto a silicon master patterned with an SU-8 photoresist and cured at 75 °C for 45 min. PDMS stamps containing micropillars were peeled from the silicon masters. The PDMS stamps were used to generate non-adhesive PEGDMA microwell arrays. PDMS stamps were placed on a PEGDMA 1,000 (20% w/v) solution containing photoinitiator Irgacure-1959 (1% w/w; Sigma) and then photo-crosslinked by exposure to UV light (350–500 nm wavelength, 5 W/cm^2^) for 45 s using the OmniCure Series 2000 curing station (EXFO). The PDMS stamp was then peeled from the substrate. The hydrogel microwell devices were sterilized in 70% isopropanol under UV for 1 h, were washed three times with phosphate buffer saline (PBS) to remove isopropanol and observed under microscope for any imperfections/air bubbles. The sterilized devices were used to generate size-controlled neurospheres and hBORGs as described below.

### Generation of neurospheres and human brain organoids (hBORGs)

Isolated NPCs were expanded in 175 mm flasks in NPC media (StemPro NSC SFM media (Gibco) supplemented with 20 ng/mL of both human recombinant FGF and EGF (Gibco)). Half of the media was exchanged every 4 days with fresh media. Cultures were kept in a humidified incubator at 37 °C and an atmosphere of 5% CO_2_. NPCs were cultured statically in suspension until they formed loose aggregates (4–7 days). To generate size controlled neurospheres, NPCs (20 × 10^6^ cells/device/50 μL NPCs media) were seeded on top of each microwell device (1 × 1 cm^2^) and were allowed to settle inside the microwells (30 min), followed by addition of NPC media. The devices were incubated at 37 °C and an atmosphere of 5% CO_2._ After 24 h, the NPC media was changed to remove floating cells. Optimum compaction of neurospheres was observed when the borders were smooth and optically translucent, as observed by light microscopy (circa 4 days). When neurospheres were formed, the media was aspirated and replaced according to the four treatments to be tested. One set of devices was replenished with NPC media only (NS); another set of devices was replenished with mixed differentiation media (DM) only (NS + DM). To test the effect of matrigel on boosting differentiation, two additional set of devices were prepared. Forty μL of matrigel (Corning) was applied on the top of the two sets of devices to cover all the neurospheres (70–80/1 × 1 cm^2^ device) and allowed to gel by incubating for 30 min at 37 °C and culture media was replaced with either fresh NPC media (NS + M) or differentiation media (NS + DM + M). Half of media was routinely replenished every other day until the hBORGs were harvested for downstream analysis.

### Cell culture of primary and immortalized microglia

HEK293T, U87MG CD4^+^ CCR5^+^ and immortalized HMC-3 microglia (ATCC CRL-3304) were grown in DMEM supplemented with 10% FCS, 1% glutamine and 1% penicillin–streptomycin. All cell lines were kept in at 37 °C and 5% CO_2_. Primary adult human microglia were obtained from Dr. Changiz Geula from Northwestern University. Briefly, microglia were isolated from the prefrontal cortex of a 71 years old Caucasian male (*postmortem* interval of 31 h). Experiments were conducted with passages between 8 and 10. Brain tissue from this patient was obtained from Northwestern University Alzheimer’s Disease Center Brain Bank (AG13854). The study was approved by the Northwestern University Institutional Review Board and conducted in accordance with the Helsinki Declaration. Written informed consent was obtained for the collection of human tissue. Culture was maintained as previously published^83^.

### Viral preparation

HIV-1 virus stocks were generated using the neurotropic proviral DNA construct pNL43-YU2-Env with enhanced green fluorescent protein (EGFP) as reporter gene. HEK293T cells (2 × 10^6^) were transfected with 3.5 μg of proviral construct and 1.5 μg of vesicular stomatitis virus G (VSV-G)-Envelope expression plasmid using 15 μL PolyJet transfection reagent (SignaGen Laboratories) as described before^55^. Viruses were collected 48 h post transfection, centrifuged at 3000* g* and filtered (0.2 μm) followed by ultracentrifugation for 60 min at 20,000 rpm (4 °C) and stored at − 80 °C until further use. Viruses were tittered using U87MG CD4^+^ CCR5^+^ permissive cells to determine the infectivity and plotted as infectious units/mL (IP/mL).

### Infection of microglia and incorporation into hBORGs

Human primary microglia and immortalized HMC3 microglial cell line (ATCC CRL-3304) were infected with HIV-1 strain NLYU2-eGFP at a multiplicity of infection (MOI) of 1.0. Mock infection was performed using equal amount of PBS. Mock and infected cells were maintained in culture until infected cells expressed EGFP (72 h p.i.). The proportion of cells expressing EGFP was estimated to be 30% using an inverted fluorescence microscope. Microglia (both mock and infected) were detached from the flasks and labeled with tracking dye CellVue Claret Far red as a second color to distinguish between the uninfected microglia (red) and infected microglia (green + red). In parallel, 2-week old hBORGs originated from NS + DM + M treatment were rinsed with PBS, and nuclei were labeled with Hoechst (1:1,000 in PBS) for 1 h. We have standardized the optimal density of microglia added to the hBORGs as 5% of total number of NPCs (1 microglia to 20 NPCs) based on previous studies on microglial density in the normal adult cortex^36,84^. Labelled infected and mock-infected microglia (1 × 10^6^ microglia/well) were added to the Hoechst-labeled hBORGs and were incubated without agitation for 24 h to allow attachment of microglia to the hBORG surface. The MG-hBORGs were then carefully transferred to a new plate with fresh differentiation media to remove unattached MGs and were maintained in culture in differentiation media for an additional 15 days. Half of the media was changed every other day and supernatants stored in − 80 °C until assayed.

### Histology and immunofluorescence

NPC-derived neurons and astrocytes were stained as described^20^ to assess the differentiation into neuronal and/or astrocytic lineages. Antibodies and concentrations were the same as described for 3D immunostaining (Table S1). Staining of the hBORGs was carried out in 4-well or 8-well chambered cover glass (LabTek, Thermo Scientific Nunc). hBORGs were transferred from the original well followed by wash with PBS and fixation in 4% paraformaldehyde overnight at 4 °C. After fixation, hBORGs were either embedded and sectioned at 10 μm on a microtome as described^85^, or followed by their immersion in 95% chilled methanol for 15 min on ice and another washing step in PBS for whole organoid staining. Permeabilization buffer was prepared by diluting Triton X-100 (0.1% v/v) in PBS. hBORGs were incubated in permeabilization buffer for 1.5 h at room temperature, followed by washing with PBST (0.1% v/v Tween20 in PBS) 3 times for 5 min, each. Blocking/dilution buffer was prepared by adding BSA (3% w/v) in permeabilization buffer. hBORGs were incubated in blocking solution for 1 h at room temperature followed by washing with PBST 3 times for 5 min each. Further, hBORGs were incubated with primary antibodies diluted in blocking buffer at 4 °C overnight followed by species-specific secondary antibodies at 4 °C overnight (Table S1). Post staining with secondary antibodies, hBORGs were washed 3 times with PBST and counterstained with 1 × HOECHST (in PBS) at 4 °C overnight and washed with PBS and immersed in glycerol for storage and imaging. Paraffin sections were hydrated in a descending series of alcohol, followed by PBS. Finally, sections were circled with a Liquid Blocker Mini Pap Pen (Life Technologies), blocked, permeabilized and stained as previously described^85^.

### Confocal microscopy and image analysis

Confocal imaging was carried out using a Z-stacking function on the Olympus FV1000 inverted confocal microscope using step size of 5–10 μm to allow visualization of the entire hBORGs and 0.5–1.5 μm for coverslips and paraffin sections. Maximum intensity Z-projections were generated using Olympus Fluoview FV10-ASW 4.2 software. For supplementary movie S1 generation, Z-stacks of the entire hBORGs were acquired and exported as mp4 format in an interval of 500 ms between. Images shown are representative of cultures generated from 3 independent experiments and from 2 independent tissue donors derived NPCs.

### Cell viability assay and image analysis

Cell viability of neurospheres and hBORGs was checked using the LIVE/DEAD staining kit (488/570, Molecular Probes, Life Technologies) as per manufacturer’s specifications. Images were taken on the inverted Olympus FV1000 microscope, with identical acquisition settings and processed using ImageJ 1.52q (National Institutes of Health, USA).

### ELISA

Levels of IL-1β and TNF-α were measured in the supernatants from MG-hBORGs containing HIV-1 and mock-infected microglia and hBORGs alone by standard sandwich ELISA using human DuoSet ELISA kit (R&D Systems) following the manufacturer’s protocol.

### Cytotoxicity

LDH activity was measured to assess cell damage/cell death in supernatants from HIV-infected MG-hBORGs compared to mock-infected MG-hBORGs by CyQUANT LDH Cytotoxicity assay kit, as per manufacturer’s protocol. Results were plotted as percentage of cytotoxicity using the following formula: [sample LDH activity-spontaneous LDH activity/Maximum LDH activity-spontaneous LDH activity] × 100.

### RNA extraction and quantitative real time PCR

RNA was isolated from hBORGs using the MirVana kit (ThermoFisher) as per manufacturer’s recommendations. The concentration and purity of the RNA were measured by NanoDrop p2000 spectrophotometer (ThermoFisher Scientific) and RNA quality by Bioanalyzer (RIN values between 8 and 9.7). cDNA was prepared from 200 to 400 ng of total RNA using a high-capacity cDNA reverse transcription kit (ThermoFisher) in 20 µL total volume reaction. Quantitative real time PCR was performed using TaqMan Universal PCR master mix and the appropriate TaqMan assays or primers (Table S2) with 2 µL of the cDNA reaction mixture. Assays were conducted using an ABI Vii7 real time PCR system in the following cycling conditions: activation of Taq DNA polymerase at 95 °C for 10 min, followed by 40 cycles of amplification at 95 °C for 15 s and 60 °C for 1 min. Results were normalized to the expression of the endogenous control Ribosomal Protein Lateral Stalk Subunit P0 (RPLP0).

### Statistical analysis

Expression level of specific transcripts in hBORGs and cells were calculated by the 2^−ΔΔCt^ method and expressed as fold change to undifferentiated NPCs or mock-infected hBORGs. Statistical analysis was performed using GraphPad Prism, version 7.0 (GraphPad Software, La Jolla, CA, USA). Statistical significance was calculated using unpaired t-test and *p* < 0.05 was considered significant.

## Supplementary information

Supplementary Information 1.

Supplementary Information 2.

Supplementary Information 3.

Supplementary Information 4.

Supplementary Information 5.

Supplementary Information 6.

Supplementary Information 7.

Supplementary Information 8.

Supplementary Information 9.
