# Supplementary figures and images for: Modeling HIV-1 neuropathogenesis using three-dimensional human brain organoids (hBORGs) with HIV-1 infected microglia
Source: Sci Rep. 2020 Sep 16;10:15209. doi: 10.1038/s41598-020-72214-0 (PMC7494890; doi:10.1038/s41598-020-72214-0)

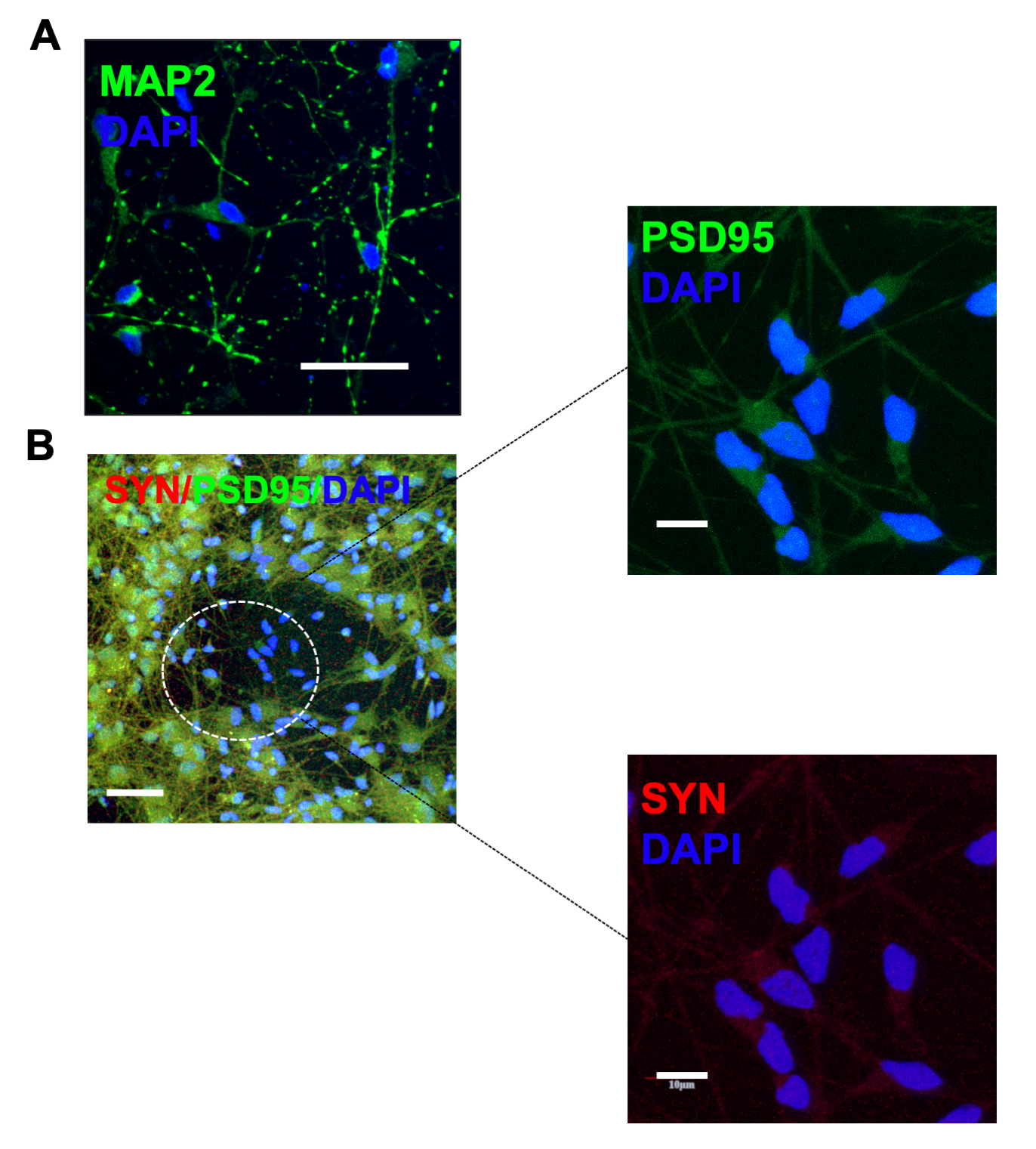

Supplement: Supplementary file 1 — Supplementary Information 1. [file 41598_2020_72214_MOESM1_ESM.tiff]

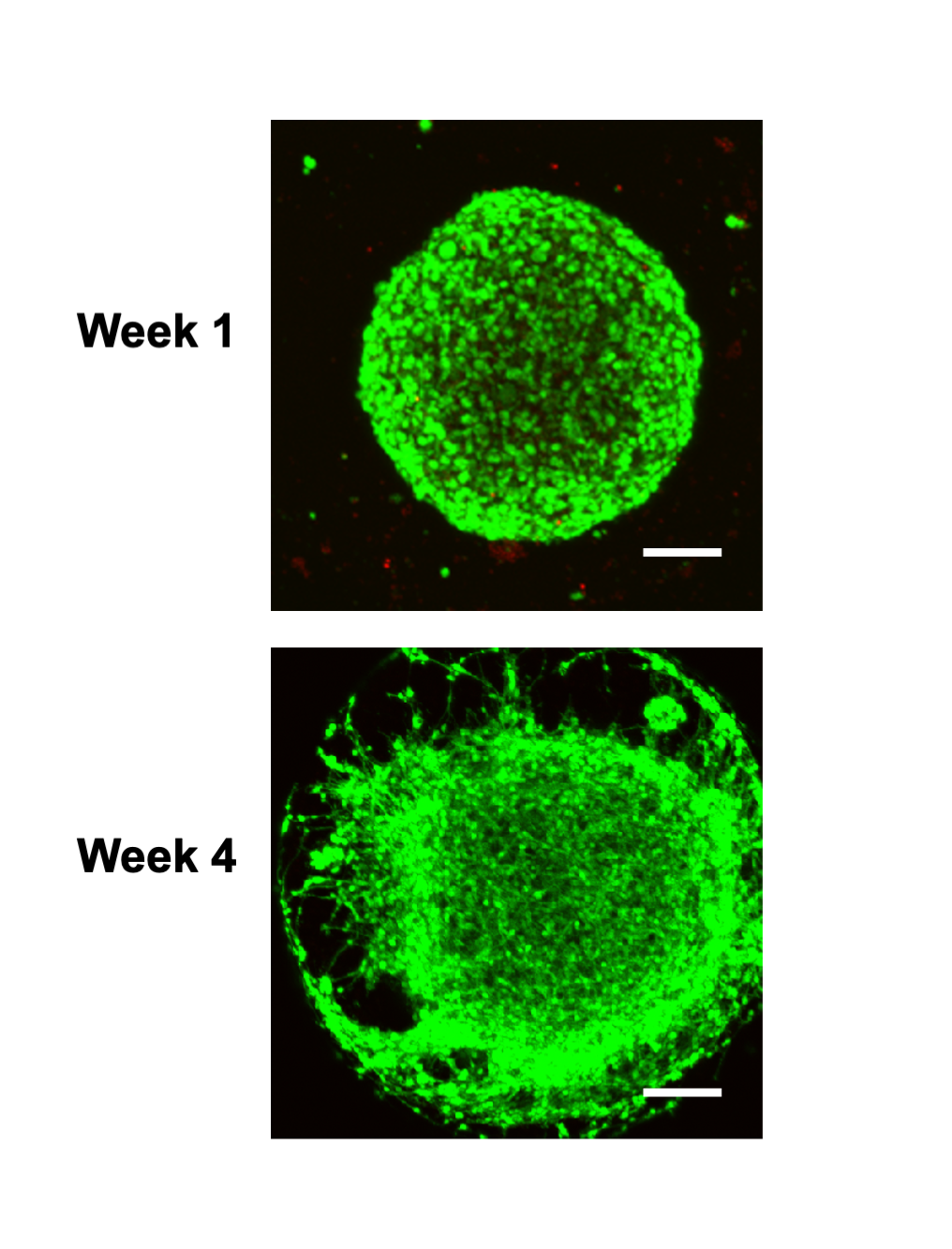

Supplement: Supplementary file 2 — Supplementary Information 2. [file 41598_2020_72214_MOESM2_ESM.tiff]

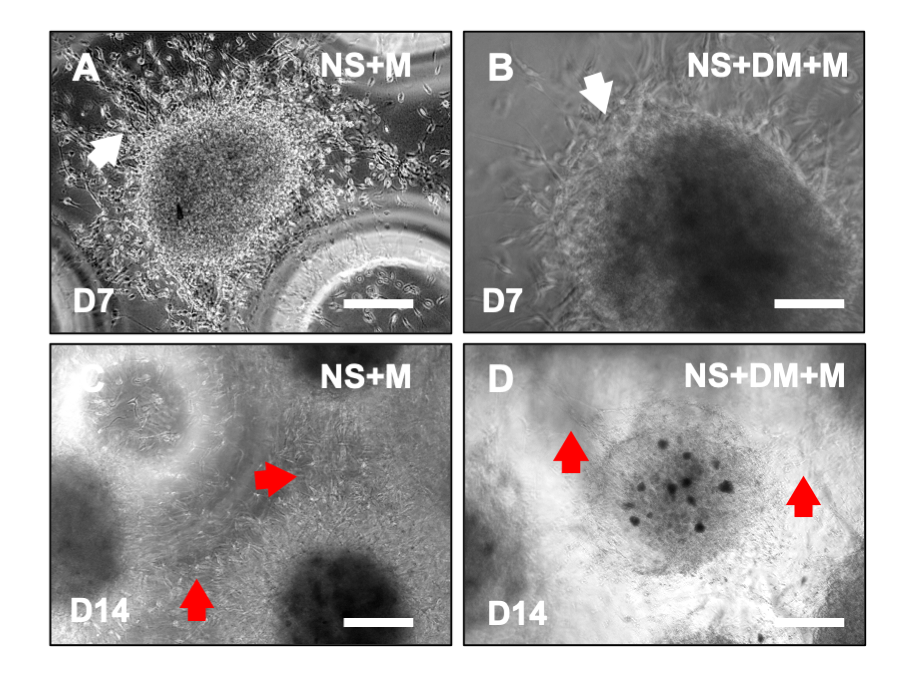

Supplement: Supplementary file 3 — Supplementary Information 3. [file 41598_2020_72214_MOESM3_ESM.tiff]

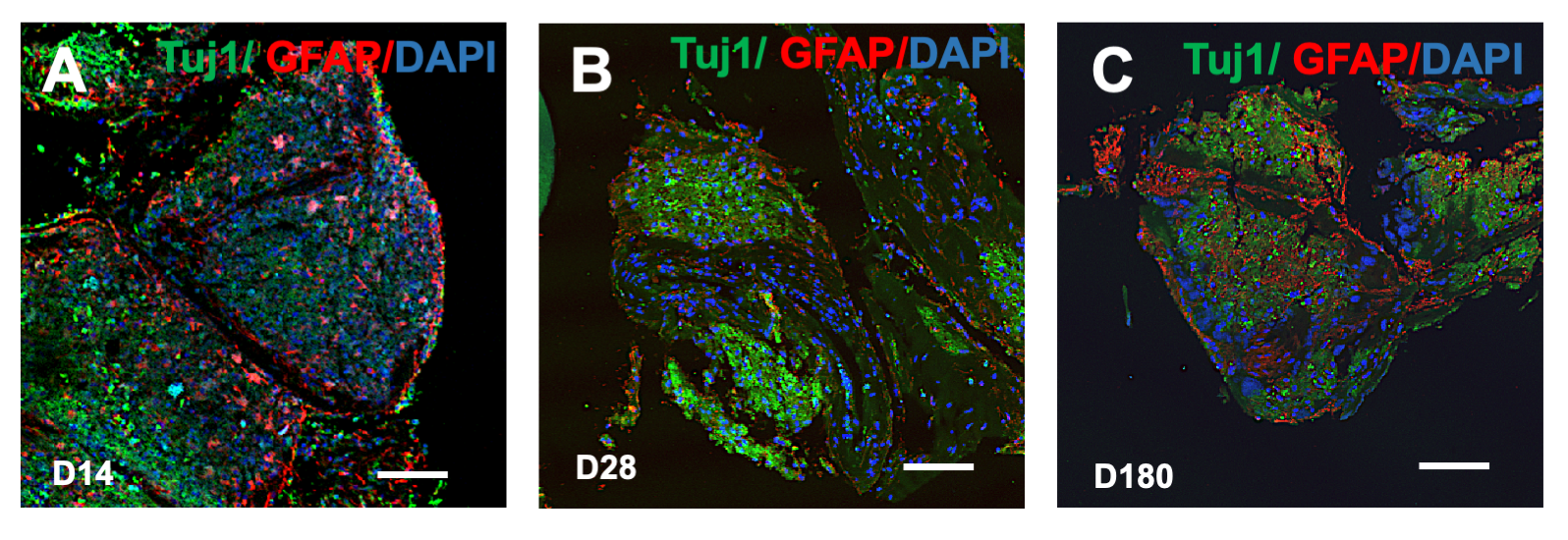

Supplement: Supplementary file 4 — Supplementary Information 4. [file 41598_2020_72214_MOESM4_ESM.tiff]

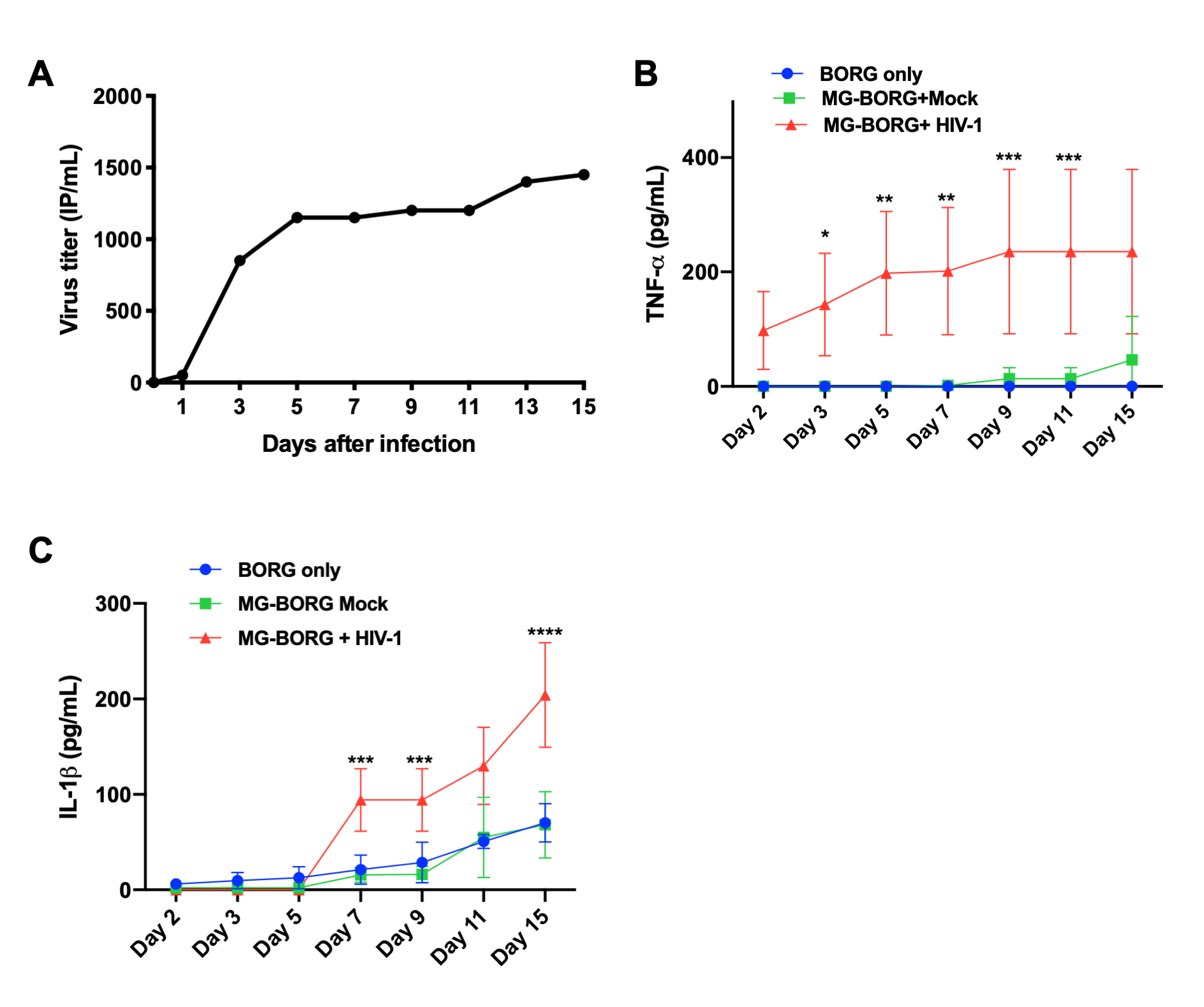

Supplement: Supplementary file 5 — Supplementary Information 5. [file 41598_2020_72214_MOESM5_ESM.tiff]
