# Supplementary material for: Modeling HIV-1 neuropathogenesis using three-dimensional human brain organoids (hBORGs) with HIV-1 infected microglia
Source: Sci Rep. 2020 Sep 16;10:15209. doi: 10.1038/s41598-020-72214-0 (PMC7494890; doi:10.1038/s41598-020-72214-0)
Supplement: Supplementary file 6 — Supplementary Information 6. [file 41598_2020_72214_MOESM6_ESM.docx]

**Modeling HIV-1 Neuropathogenesis Using Three-dimensional Human Brain Organoids (hBORGs) with HIV-1 Infected Microglia**

*Roberta S. dos Reis^1^, Shilpa Sant^2,3*^, Hannah Keeney^3^, Marc C. E. Wagner^1^ and Velpandi Ayyavoo^1^*^,*^

^1^Department of Infectious Diseases and Microbiology, Graduate School of Public Health, University of Pittsburgh, Pittsburgh, PA 15261, USA; ^2^Department of Pharmaceutical Sciences, School of Pharmacy, McGowan Institute for Regenerative Medicine, UPMC Hillman Cancer Center, ^3^Department of Bioengineering, Swanson School of Engineering, University of Pittsburgh, Pittsburgh, PA 15261, USA.

*Corresponding authors:

Velpandi Ayyavoo, PhD

[velpandi@pitt.edu](mailto:velpandi@pitt.edu)

Shilpa Sant, PhD

[shs149@pitt.edu](mailto:shs149@pitt.edu)

**Supporting information**

**Figure S1. Characterization of neuronal population in 2D mixed primary cultures.** NPCs were differentiated to neurons for 6 weeks according to protocol and characterized by immunofluorescence for expression of **(A)** MAP2 (green) as mature neuronal marker; **(B)** Synaptophysin (SYN, red) and PSD95 (green) as markers of synaptic integrity. Nucleus was stained with DAPI (blue). Scale bar panels A and B, 50 μm; Scale bar insets, 10 μm.

**Figure S2. Determination the viability of neurospheres before differentiation by live/dead staining.** NPCs were seeded on hydrogel devices to form uniform size neurospheres. Cell viability in neurospheres was assessed by live/dead assay followed by confocal microscopy. Images represent live cells (green) and dead cells (red). Scale bar is 100 μm.

**Figure S3. Morphological changes of hBORGs in culture over time.** Bright field images of neurospheres (NS) with and without differentiation media (DM) with matrigel (M) were captured on day 7 and 14. White arrowheads point to neurites extension that are seen after 7 days NS+M culture **(A)** and NS+DM+M **(B)**. Red arrowheads point to the connection among the neurospheres that were seen after 14 days in NS+M culture **(C)** and NS+DM+M condition **(D)**. Scale bar is 200 μm.

**Figure S4. Differentiation of hBORGs in culture over time.** hBORGs were harvested after 14 (A), 28 (B) or 180 days (C) post differentiation, fixed and sectioned. Sections were immunostained with anti-TuJ-1(green) and anti-GFAP (red) and imaged. Nucleus was stained with DAPI (blue). Scale bar is 100 μm.

**Figure S5**. **Microglial cell line, HMC3 incorporated in hBORGs supports HIV-1 replication and exhibits inflammatory response to infection. (A)** Cumulative virus titer in supernatants of MG-hBORGs generated from a representative organoid (N=3) containing HIV-1 infected HMC3 cells on day 15 post culture by qRT-PCR. Cumulative levels of TNF-α (**B**) and IL-1β (**C**) released from HIV-infected, mock infected MG-hBORGs and hBORGs measured by ELISA. Figure represents results from one of three independent experiments (N=3) from a representative NPC donor. *, p<0.05.

**Supplementary movie S1.**  Microglia cells expressing EGFP incorporated into hBORGs 3 days post co-culture. MG-hBORGs were fixed and immunostained for MAP2 (orange). Movie depicts stacks through the entire organoid recorded as mp4 with an interval of 500 milliseconds between frames**.**
