# Supplementary material for: Modeling HIV-1 neuropathogenesis using three-dimensional human brain organoids (hBORGs) with HIV-1 infected microglia
Source: Sci Rep. 2020 Sep 16;10:15209. doi: 10.1038/s41598-020-72214-0 (PMC7494890; doi:10.1038/s41598-020-72214-0)
Supplement: Supplementary file 8 — Supplementary Information 8. [file 41598_2020_72214_MOESM8_ESM.docx]

**Table S1: Antibodies resources**

| **Antibody to:** | **Host species** | **Dilution** | **Manufacturer** |
| --- | --- | --- | --- |
| βIII-Tubulin (clone Tuj-1) | mouse | 1:200 | R&D Systems |
| MAP2 (microtubule associated protein 2) | mouse | 1:200 | Merck |
| GFAP (glial fibrillary acidic protein) | chicken | 1:500 | Abcam |
| SOX2 | rabbit | 1:200 | Bioss |
| Nestin | mouse | 1:200 | R&D Systems |
| NeuN | rabbit | 1:200 | Proteintech |
| VGlut1 | rabbit | 1:200 | Sigma-Millipore |
| VGAT | rabbit | 1:100 | Proteintech |
| TH | mouse | 1:100 | Proteintech |
| PSD95 | rabbit | 1:100 | Abcam |
| Synaptophysin | mouse | 1:100 | Novus Biologicals |
