# Supplementary material for: Modeling HIV-1 neuropathogenesis using three-dimensional human brain organoids (hBORGs) with HIV-1 infected microglia
Source: Sci Rep. 2020 Sep 16;10:15209. doi: 10.1038/s41598-020-72214-0 (PMC7494890; doi:10.1038/s41598-020-72214-0)
Supplement: Supplementary file 9 — Supplementary Information 9. [file 41598_2020_72214_MOESM9_ESM.docx]

**Table S2: RT-PCR primer sets and probes used in this study**

| **mRNA** | **Description** | **5’-3’ sequence** |
| --- | --- | --- |
| Nestin | Assay ID | Applied Biosystems TaqMan® Gene Expression Assays ID: [Hs04187831_g1](https://www.thermofisher.com/taqman-gene-expression/product/Hs04187831_g1?CID=&ICID=&subtype=) |
| βIII-Tubulin | Assay ID | Applied Biosystems TaqMan® Gene Expression Assays ID: Hs00801390_s1 |
| MAP2 | Assay ID | Applied Biosystems TaqMan® Gene Expression Assays ID: Hs00258900_m1 |
| GFAP | Assay ID | Applied Biosystems TaqMan® Gene Expression Assays ID: [Hs00909233_m1](https://www.thermofisher.com/taqman-gene-expression/product/Hs00909233_m1?CID=&ICID=&subtype=) |
| Iba1 (AIF1) | Assay ID | Applied Biosystems TaqMan® Gene Expression Assays ID:Hs00610419_g1 |
| SS-Gag | Primer F | TCTCTAGCAGTGGCGCCCGAACA |
|  | Primer R | TCTCCTTCTAGCCTCCGCTAGTC |
|  | Probe | CGGGAG TACTCACCAGTCGCCGCCCCTCGCC CTCCCG |
| MS-Gag | Primer F | CTTAGGCATCTCCTATGGCAGGAA |
|  | Primer R | TTCCTTCGGGCCTGTCGGGTCCC |
|  | Probe | GGGCCTTCTCTATCAAAGCAACCCACCTCCAGGCCC |
